# The Effects of Pesticides on Queen Rearing and Virus Titers in Honey Bees (*Apis mellifera* L.)

**DOI:** 10.3390/insects4010071

**Published:** 2013-01-04

**Authors:** Gloria DeGrandi-Hoffman, Yanping Chen, Roger Simonds

**Affiliations:** 1 Carl Hayden Bee Research Center, USDA-ARS, 2000 East Allen Road, Tucson, AZ 85719, USA; 2 Beltsville Bee Research Laboratory, 10300 Baltimore Avenue, Bldg. 476 BARC-East, Beltsville, MD 20705, USA; E-Mail: Judy.Chen@ARS.USDA.GOV; 3 Agricultural Marketing Service, National Science Laboratory, 801 Summit Crossing Place, Suite B, Gastonia, NC 28054, USA; E-Mail: Roger.Simonds@AMS.USDA.GOV

**Keywords:** deformed wing virus, Black queen cell virus, chlorpyrifos, boscalid, pyraclostrobin

## Abstract

The effects of sublethal pesticide exposure on queen emergence and virus titers were examined. Queen rearing colonies were fed pollen with chlorpyrifos (CPF) alone (pollen-1) and with CPF and the fungicide Pristine^®^ (pollen-2). Fewer queens emerged when larvae from open foraging (*i.e.*, outside) colonies were reared in colonies fed pollen-1 or 2 compared with when those larvae were reared in outside colonies. Larvae grafted from and reared in colonies fed pollen-2 had lower rates of queen emergence than pollen-1 or outside colonies. Deformed wing virus (DWV) and black queen cell virus were found in nurse bees from colonies fed pollen-1 or 2 and in outside colonies. The viruses also were detected in queen larvae. However, we did not detect virus in emerged queens grafted from and reared in outside colonies. In contrast, DWV was found in all emerged queens grafted from colonies fed pollen-1 or 2 either reared in outside hives or those fed pollen-1 or 2. The results suggest that sublethal exposure of CPF alone but especially when Pristine^®^ is added reduces queen emergence possibly due to compromised immunity in developing queens.

## 1. Introduction

Populations of honey bees throughout the world have been declining for more than a decade. In the past 20 years, the number of colonies in the U.S. fell from 4.2 million to 2.4 million [1,2]. Colony losses are attributed to a myriad of causes including exposure to pesticides. Honey bees are highly sensitive to pesticides because they have relatively few genes encoding detoxiﬁcation enzymes (e.g., cytochrome P450 monooxygenases (P450s), glutathione-S-transferases, and carboxylesterases) [3,4]. Colonies of honey bees often are exposed to pesticides when foragers gather contaminated nectar and pollen in the field, and return with it to the hive where it is stored and shared among nestmates.

Colony losses from lethal exposure to insecticides are easy to diagnose because a large number of dead bees usually are found on the bottom or near the entrance of the hive. However, sublethal exposures might have delayed and extended effects on colony growth and survival. There might be a loss of adult bees and brood that is not great enough to kill the colony outright, but instead weakens it so that the hive either is robbed by other bees or dies over winter. Pesticides also can affect immunity and make colonies more vulnerable to loss from disease-causing agents (see references in [5]). Recent studies show an association between spore numbers of the intracellular microsporidian parasite *Nosema apis* and *N*. *ceranae* in worker bees and pesticide exposure [6,7,8,9].

The most frequently found insecticides in colonies are those used for Varroa mite control (fluvalinate and coumaphos). In addition, chlorpyrifos (CPF) often is found in pollen stores [10]. Fungicides also are commonly detected in stored pollen, and often account for most of the pesticide contamination in bee bread (a fermented mixture of pollen and nectar stored in the hive). High levels of fungicides occur in pollen because these compounds are applied during bloom.

CPF and other organophosphate (OPs) insecticides are inhibitors of acetylcholinesterase (AChE) [11]. There is increasing evidence however, that OPs have biochemical targets other than AChE [12]. For example, OPs may disrupt metabolism by altering the expression or function of adenylyl cyclase or G-protein-coupled receptors and G-proteins involved in the adenylyl cyclase signaling cascade in neuronal as well as in peripheral tissues [13]. Many OPs also alter immune functions by oxidative damage, metabolism modifications and stress-related immunosuppression [14].

Unlike insecticides that often target neural function, fungicides can affect nucleic acids and protein synthesis, cell membrane structure and function, signal transduction, respiration, mitosis and cell division [15]. The fungicide used in this study (Pristine^®^ BASF, Research Triangle Park, NC, USA) contains boscalid and pyraclostrobin. Both of these compounds affect respiration by binding to succinate ubiquinone reductase (also called Complex II) or cytochrome bc1 (Complex III) in the electron transport chain of the mitochondria [16,17,18,19]. Fungicides that compromise mitochondrial function might also suppress immunity because innate immune signaling is driven by basic host metabolic functions, such as oxygen consumption, ATP production and possibly biosynthetic pathways that depend on mitochondrial activity and fitness [20].

Pollen collected by bees that is contaminated with CPF and fungicides can persist at sublethal levels in colony food stores and possibly cause extended periods of immunosuppression among immature and adult bees. As such, colony losses attributed to viruses actually might be downstream effects of sublethal exposure to pesticides and/or fungicides. Persistent sublethal exposure might also reduce the likelihood that a colony can successfully rear a replacement queen especially if pathogens such as Black queen cell virus (BQCV) are present. In a pilot study, we found that less than half of the colonies we fed pollen contaminated with CPF and fungicides including boscalid and pyraclostrobin were able to rear new queens. Colonies that lose their queen and cannot rear a new one perish.

The purpose of this study was to determine the effects of feeding pollen contaminated in the field with CPF alone and with added fungicide on queen emergence and virus titers. We chose this combination of pesticides because we commonly detect CPF in almond pollen collected by honey bees, and Pristine^®^ is often sprayed during bloom especially in almond growing regions that experience wet weather during bloom. The occurrence of viruses and differences in titers between nurse bees and developing and emerged queens were used as a measure of the possible effects of pesticide contamination on immune function.

## 2. Experimental Section

All experiments were conducted at the Carl Hayden Bee Research Center from July through October of 2011. All colonies were comprised of Italian bees (*Apis mellifera ligustica)* and headed by commercially produced and mated European queens (Koehnen and Sons Inc., Glenn, CA, USA). Five frame nucleus colonies were used as sources for both open foraging larvae and for queen rearing colonies. The nucleus colonies were located in the apiary adjacent to the Bee Center. These colonies contained 3,000–4,000 bees with 2–3 frames of brood. Open foraging colonies collected pollen from native desert vegetation. These colonies are hereafter referred to as “outside” colonies.

### 2.1. Pollen Collection in Almond Orchard

Pollen traps were placed at the entrance of colonies located in blooming almond orchards at Paramount Farms in Lost Hills, California, USA. This site was chosen because fungicides are not sprayed during bloom and orchards are large enough to minimize the chances of drift from other sites. Chlorpyrifos (CPF) was applied to the orchard prior to bloom as Lorsban Advanced (40.18% AI) at the rate of 0.5 gals per acre on 13 January 2011 (dormant treatment) in combination with Supreme Spray Oil at 2 gal per acre. Almond pollen was collected over the 3 week bloom period beginning in February, 2011. The pollen was removed from the traps weekly and shipped frozen overnight to the Carl Hayden Bee Research Center, Tucson, AZ, USA. The pollen was kept in a −20 °C freezer until fed to the bees.

### 2.2. Application of Fungicide to the Pollen

The pollen collected in the almond orchards was ground to a powder using a coffee grinder (Mr. Coffee model 1DS77, Sunbeam, Boca Raton, FL, USA). The pollen (350 g) was spread evenly on 0.26 m^2^ trays. Pristine^®^ (1.6630 g) was added to 3.78 L of distilled water. The active ingredients in Pristine^®^ (boscalid and pyraclostrobin) comprise 38% of the formulation (boscalid = 25.2% and pyraclostrobin = 12.8%). Therefore a target concentration of 7,800 ppb of Pristine^®^ was needed to obtain about 3,000 ppb of active ingredients (boscalid = 1,966 ppb and pyraclostrobin = 998 ppb). The Pristine^®^ and water mixture was applied to the pollen using an Ace Home and Garden 2 gallon Sprayer-Plus (Ace Hardware, Oak Brook, IL, USA). The sprayer was calibrated to deliver liquid at a rate of 10.5 mL of liquid/sec. Pollen in the “chlorpyrifos” treatment was sprayed with distilled water alone. The pollen was removed from the tray, mixed in plastic bags and returned to the −20 °C freezer until it was fed to the bees.

### 2.3. Pesticide Analysis

Samples of pollen sprayed with distilled water alone (pollen-1) and with Pristine^®^ (pollen-2) were analyzed for 174 different agrochemicals by the USDA-AMS-National Science Laboratory (NSL) in Gastonia, NC. Two samples of pollen-1 were analyzed for pesticides and 3 samples of pollen-2. In addition, samples of bee bread from three colonies fed pollen-1 and samples from five colonies fed pollen-2 also were analyzed. Samples of nurse bees tending queen cells in colonies fed pollen-1 or pollen-2, queen larvae, and royal jelly also were analyzed (2 samples from each colony). Samples were extracted for pesticide residue analysis using an official pesticide extraction method (AOAC 2007.01, also known as the QuEChERS method) and analyzed by gas chromatography and liquid chromatography coupled with mass spectrometry detection (GC/MS, GC/MS/MS, LC/MS/MS). Quantification of pesticide residues was performed using external calibration standards prepared from certified standard reference material. The NSL is ISO 17025 accredited to perform pesticide residue analysis.

### 2.4. Feeding Pollen to Bees

Colonies were placed in an enclosed flight area (EFA) at the Carl Hayden Bee Research Center in Tucson, AZ. The EFA is divided into 10 separate sections that are 1.93 m wide, 8.25 m long and 4.14 m high. Each section is the same size and bees cannot fly between the sections. A nucleus colony was established in each section. All colonies were comprised about 3,000 adult bees and a laying queen. The hives contained frames with foundation, and bees were fed sugar syrup to draw comb. When the drawn comb contained larvae, pollen-1 or pollen-2 was fed *ad libitum* by placing pollen at the hive entrances. There were 4 colonies fed pollen-1 and 5 colonies fed pollen-2. Colonies were fed either pollen-1 or pollen-2 for 4 weeks before queens were grafted and during the periods when queens were reared. This procedure insured that the queen cells evaluated in the study were tended by nurse bees reared entirely on the pollen we fed.

### 2.5. Rearing Queens

Larvae (<36 h old) were grafted from worker cells into queen cups and reared into queens using the procedures described in Laidlaw [21]. Colonies used for queen rearing were made queenless for 24 h before queen cups containing larvae were introduced. Ten queen cups with larvae were placed in the center of each colony and combs with bees and brood were placed on either side. The cells remained in the colony until queens were within 48 h of emergence. At that time, the cells were removed and placed in individual vials in an environmental room with a temperature of 32–34 °C and 50% humidity. Each vial had a small piece of “queen candy” (a mixture of powdered sugar, honey and water formed into a paste) that the emerged queen could feed on while in the vial. The cells were checked daily for emerged queens.

In Experiment 1, larvae grafted from outside colonies were reared into queens in colonies fed pollen-1 or 2 in the EFA or in outside colonies. The outside colonies used for rearing queens were different from those used as sources for the grafted larvae. Groups of 10 queen cups with larvae were placed in each of 4 colonies fed pollen-1, the 5 colonies fed pollen-2, or in 3 colonies outside the EFA.

In Experiment 2, a bar with 10 queen cups containing larvae taken from each colony fed either pollen-1 or -2 were placed back in same colony from which they were grafted. A second set of larvae from these colonies (5 larvae per treatment colony) were transferred into queen cups on the same bar and reared in outside colonies. Ten larvae (5 from each treatment group) were reared in each of 4 outside colonies. The outside colonies were different from those used in Experiment-1.

### 2.6. Protein Analysis

Frames with sealed brood from colonies in the EFA fed pollen-1 or 2 (4 colonies of each) and from the 4 hives outside the EFA that were used for queen rearing were placed in screen cages in an incubator until the brood emerged. Frames were selected that had large amounts of stored pollen and nectar so that the bees could feed on it until they were sampled. Bees were marked on the day they emerged with a dot of paint on their thorax. Seven days after emergence, the bees were removed from the frame and their hemolymph was sampled (5 bees per treatment).

Soluble protein in pollen and bee bread also was estimated using three random samples of pollen-1 and -2 as fed to the bees and three samples of bee bread from two colonies each that were fed either pollen-1 or-2. Three samples of bee bread also were taken from the outside colonies used for queen rearing. Not all colonies had sufficient stores of bee bread for protein analysis.

Hemolymph was collected by inserting a 20 μL capillary tube (that had been heated and pulled to a needle-sharp point) into the right lateral portion of the thorax near the point of attachment of the wings. Additional hemolymph was collected by inserting the same tube into the membrane of the abdomen between the abdominal tergites. Hemolymph was collected within 1–2 h after bees were sampled from the frames. Approximately 1–5 μL of hemolymph was collected per bee. Hemolymph samples were stored at −20 °C until analysis.

Hemolymph samples were prepared for analysis of soluble protein concentration by adding a 1.0 μL sample to 0.9 μL of PBS. Pollen and bee bread were prepared for analysis by mixing a 20 mg sample with 1,000 μL of phosphate buffer solution (PBS). The mixture was vortexed for 10 s. and centrifuged at 8,500 rpm for 1 min. Ten microliter samples of the supernatant were placed in wells of a 96 well flat bottom EIA/RIA polystyrene plate. Each sample was replicated in three wells.

Total soluble protein concentrations in pollen, bee bread, and hemolymph samples were estimated using a Quick Start Bradford Protein Assay Kit 2 (#500-0202, Bio-Rad Laboratories, Hercules, CA, USA) [22,23]. Standard curves to estimate soluble protein concentration in the samples were prepared using bovine serum albumin (BSA). Protein absorbance was measured at 595 nm using a Biotek Synergy HT spectrophotometer.

### 2.7. Virus Analysis

Nurse bees (2–3 from each colony for a total of 10–15 bees) were sampled while tending queen cells. Three queen larvae and an emerged queen also were sampled from each colony. All samples were placed in liquid nitrogen and stored at −70 °C until analysis for viruses.

*RNA Extraction. *Individual bees were subjected to RNA extraction. Each bee was ground manually in a 1.5 mL eppendorf tube using a pestle that fits into the tube. Total RNA was extracted from each bee using Trizol reagent according to the manufacturer’s instructions (RNA extraction kit; Invitrogen, Carlsbad, CA, USA). The resultant RNA pellets were re-suspended in nuclease-free water in the presence of Ribonuclease Inhibitor (Invitrogen, Carlsbad, CA, USA). The quantity and purity of RNA were measured by NanoDrop Spectrophotometer (NanoDrop Technologies, Wilmington, DE, USA). RNA samples were stored at −80 °C.

SYBR-Green real-time quantitative RT-PCR (qRT-PCR) was performed for detection and quantification of viruses. RNA samples extracted from bees were subject to RT-PCR analysis for seven viruses including: Acute bee paralysis virus (ABPV), BQCV, Chronic bee paralysis virus (CBPV), DWV, Israeli acute paralysis virus (IAPV), Kashmir bee virus (KBV) and Sacbrood bee virus (SBV). The primer sets used for RT-PCR amplification of the viruses have been reported previously [24,25]. qRT-PCR was performed using Stratagene’s Mx3005P™ Real-Time PCR System operated by MxPro qPCR software. qRT-PCR was carried out in a 50-µL reaction volume containing 25 µL of 2× Brilliant^®^ SYBR^®^ Green qRT-PCR Master Mix (Stratagene, La Jolla, CA, USA), 0.4 µM each of forward and reverse primers, and 200 ng of template RNA. The thermal profile parameters consisted of one cycle at 50 °C for 30 min, one cycle at 95 °C for 3 min followed by 30 cycles of 95 °C for 30 s, 55 °C for 1 min, and 72 °C for 30 s. Negative controls (no reverse transcriptase and no template) were included in each run of the reaction. The positive control was purposely not included in the reaction in order to avoid any potential contamination. After amplification, a melting curve analysis was performed to determine the specificity of the PCR products. The PCR products were incubated for 1 min at 95 °C, ramping down to 55 °C at a rate of 0.2 °C/s. The dissociation curve was constructed using 81 complete cycles of incubation where the temperature was increased by 0.5 °C/cycle, beginning at 55 °C and ending at 95 °C. The expression of a housekeeping gene, β-actin, in each sample also was measured for normalization of real time qRT-PCR results using a pair of primers published previously [24].

The output of qRT-PCR assays of each virus was interpreted by using the comparative Ct method (ΔΔCt Method). The virus level was quantified based on the value of the cycle threshold (Ct) which represents the number of cycles needed to generate a fluorescent signal above a predefined threshold and therefore is inversely proportional to the concentration of the initial target that has been amplified. For each detected virus, the average Ct value (ΔCt) of a virus was normalized using the Ct value corresponding to the β-actin following the formula: ΔCt = (Average Ct_DWV_) − (Average Ct_β-actin_). The group with the lowest level of the minimal virus level was chosen as a calibrator. The ΔCt value of each group was subtracted by ΔCt value of the calibrator to yield ΔΔCt. The virus concentration in each group was calculated using the formula 2^−ΔΔCt^ and expressed as an n-fold difference relative to the calibrator.

### 2.8. Statistical Analysis

The protein concentration measured in the 5 bees per colony (pollen-1, pollen-2 and outside colonies) was summed to generate a single value for the colony. These colony values were used in a one way analysis of variance to compare hemolymph of 7-day old worker bees. The average protein concentrations in pollen and bee bread were compared using t-tests. Queen emergence in colonies fed pollen-1 or pollen-2 were compared with outside colonies in Experiment-1 using a Chi-square test. The number of queen cells that were sealed and that had emerged queens in the outside colonies were used as expected values. In Experiment-2, comparisons were made between the number of queen cells with larvae from colonies fed either pollen-1 or pollen-2 that were sealed and had emerged queens when reared in those colonies versus outside colonies. Comparisons were made using a Chi-square test with numbers of cells that were sealed and had emerged queens in the outside colonies as expected values. The number of nurse bees, queen larvae and virgin queens testing positive for DWV or BQCV was compared between those sampled from outside colonies and in the EFA using Chi-square test where values for the outside colonies were the “expected”. Differences in fold increases of DWV and BQCV in queen larvae and virgin queens were determined among the treatments using analysis of variance.

## 3. Results

### 3.1. Pesticide Analysis

**Figure 1:**
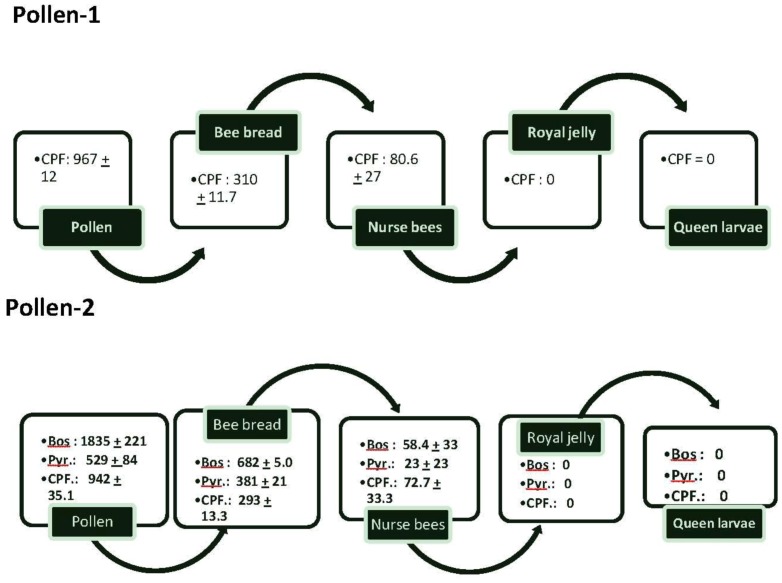
The flow of average amounts (±SEM) of chlorpyrifos (CPF), boscalid (Bos) and Pyraclostrobin (Pyr) from pollen into bee bread, nurse bees feeding on the bee bread, royal jelly secreted by the nurse bees and queen larvae feeding on the royal jelly. Two samples of pollen-1 were analyzed for pesticides and 3 samples of pollen-2. In addition, samples of bee bread from 3 colonies fed pollen-1 and 5 from those fed pollen-2 also were analyzed. Samples of nurse bees tending queen cells in colonies fed pollen-1 or pollen-2, queen larvae, and royal jelly also were analyzed (2 samples from each colony). In all cases, multiple samples from each colony were pooled for analysis.

Pollen collected in almond orchards and referred to as “pollen-1” had an average of 967 ± 12 ppb of CPF (Figure 1). The concentrations decreased so that there was only about one-third of the amount of CPF in bee bread. Nurse bees had less than 25% of the concentration of CPF found in bee bread; there was no CPF in the royal jelly or queen larvae.

In pollen-2, the two components of Pristine^®^—boscalid and pyraclostrobin were detected at 1,835 ± 221 and 529 ± 84.2 ppb respectively. CPF was detected at 942 ± 35.1. As in pollen-1, CPF concentrations in bee bread were less than those found in pollen as were concentrations of boscalid and pyraclostrobin. The compounds also were found in nurse bees tending the queen cells at 72.7 ± 33.3 ppb for CPF, 58.4 ± 33.1 for boscalid and 23.1 ± 23.1 for pyraclostrobin. CPF and the components in Pristine^®^ were not found in royal jelly or in the queen larvae.

### 3.2. Protein Concentrations

The concentrations of soluble protein in the pollen and bee bread made from it were not significantly different between pollen-1 and pollen-2 (F_3,14_ = 0.88, *p* = 0.476). We combined pollen-1 and -2 samples and determined the overall protein concentration in the almond pollen to be 586.0 ± 47.4 µg/mL and in bee bread to be 653 ± 20.8 µg/mL. These averages were not significantly different (t_6_ = 1.29, *p* = 0.243). There also was no significant difference in protein concentrations between bee bread sampled from outside colonies (average = 676.9 ± 26.5 µg/mL) and those in the EFA (F_2,12_ = 0.23, *p* = 0.8, d.f. = 2, 12). The protein concentration in the hemolymph of 7-day old workers fed pollen-1, 2 or open foraging outside the EFA was not significantly different (F_2,8_ = 0.89, *p* = 0.448) (sum of 5 bees per treatment: pollen-1 = 1,973 ± 234 µg/mL, pollen-2 = 1,685 ± 350 µg/mL, outside = 1,447 ± 102 µg/mL).

### 3.3. Queen Emergence

**Figure 2:**
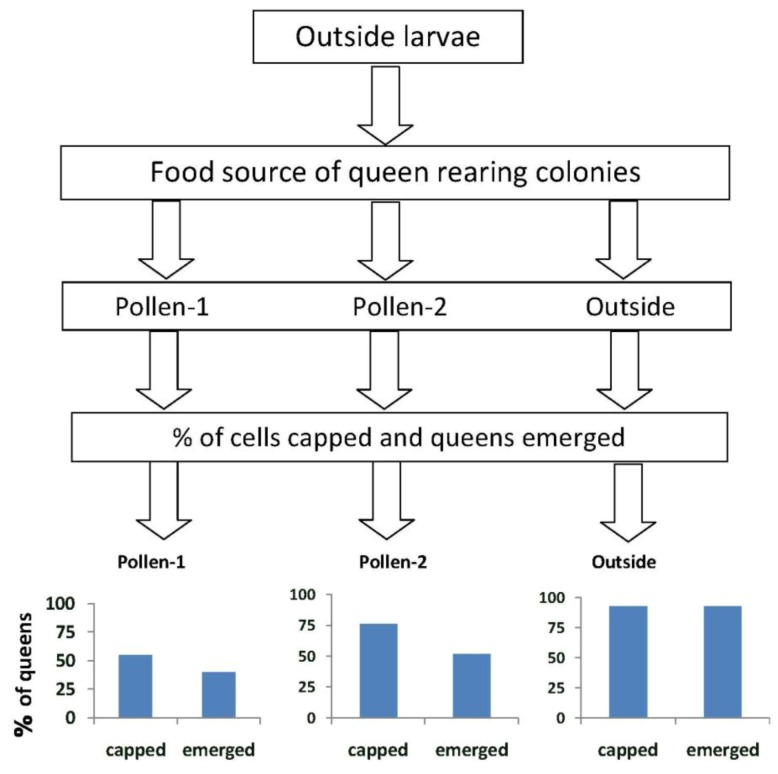
The percentages of queen cells that were capped and queens that emerged when larvae from open foraging (“outside”) colonies were reared as queens in outside colonies or in hives in an enclosed flight area that were fed pollen with chlorpyrifos alone (pollen-1) or with added Pristine^®^ fungicide (pollen-2). Percentages of capped queen cells and emerged queens in outside colonies were significantly higher than when the larvae were reared as queens in colonies fed pollen-1 (55% survival to capped stage; *X*^2^ = 12.3, *p* < 0.0001 and 40% emergence; *X*^2^ = 21.0, *p* ≤ 0.0001 ) or pollen-2 (76% survival to capped stage *X*^2^ = 4.03, *p* = 0.046; and 51.8% emergence *X*^2^ = 13.1, *p* < 0.0001). The percentage of larvae from outside colonies that survived to the capped brood stage in colonies fed pollen-2 was greater than in pollen-1 (76% and 55% respectively; *X*^2^ = 4.55, *p* = 0.03). The percentages that emerged as queens however, did not differ (pollen-1 = 40%, pollen-2 = 51.8%; *X*^2^ = 1.29, *p* = 0.255).

When larvae from outside colonies were reared into queens in outside colonies (Experiment 1), 93% survived to the capped brood stage and 93% emerged (Figure 2).These percentages were significantly higher than those when the larvae were reared into queens in colonies fed pollen-1 (55% survival to capped stage; *X*^2 ^= 12.3, *p* < 0.0001 and 40% emergence; *X*^2^ = 21.0, *p* ≤ 0.0001) or pollen-2 (76% survival to capped stage *X*^2^ = 4.03, *p* = 0.046; and 51.8% emergence *X*^2^ = 13.1, *p* < 0.0001). More larvae from outside colonies survived to the capped brood stage in colonies fed pollen-2 compared with pollen-1 (76% and 55% respectively; *X*^2^ = 4.55, *p* = 0.03). The percentages that emerged as queens however did not differ (pollen-1 = 40%, pollen-2 = 51.8%; *X*^2^ = 1.29, *p* = 0.255).

**Figure 3:**
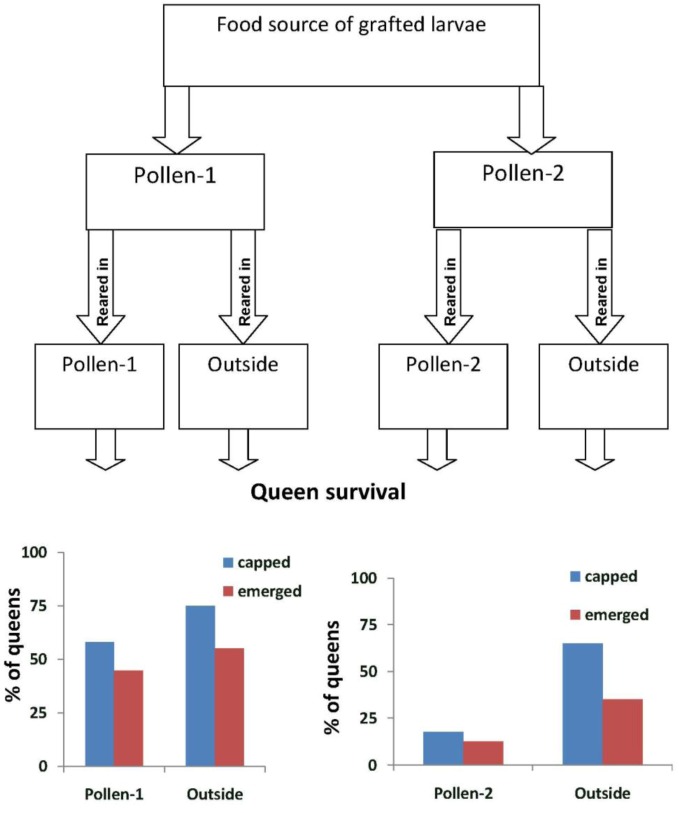
The percentage of queen cells that were capped and had emerged queens when larvae were grafted from colonies in an enclosed flight area (EFA) that were fed pollen with chlorpyrifos alone (Pollen-1), or with chlorpyrifos and Pristine^®^ fungicide (Pollen-2). The larvae were reared into queens in the colonies fed pollen-1 or 2 or in open foraging colonies outside the EFA (Outside). Percentages of larvae grafted from colonies fed pollen-1 that survived to the capped stage did not differ between those reared in colonies fed pollen-1 (57.9%) or in outside colonies (75%) (*X*^2^ = 1.65, *p* = 0.198). The percent that emerged also did not differ (*X*^2^ = 0.55, *p* = 0.457). Significantly fewer larvae from colonies fed pollen-2 and reared in those colonies survived to the capped stage (*X*^2^ = 13.54, *p* < 0.0001) and emerged as queens compared with those reared in outside colonies (*X*^2^ = 4.21, *p* = 0.04). More larvae survived to the capped brood stage and emerged as queens when grafted from and reared in colonies fed pollen-1 compared with pollen-2 (capped brood: *X*^2^ = 13.6, *p* < 0.0001; emerged queens: *X*^2^ = 10.0, *p* = 0.002).

In Experiment-2, the percentage of larvae grafted from colonies fed pollen-1 that survived to the capped stage did not differ between those reared in colonies fed pollen-1 (57.9%) or in outside colonies (75%) (*X*^2^ = 1.65, *p* = 0.198) (Figure 3). The percent that emerged also did not differ (*X*^2^ = 0.55, *p* = 0.457). Significantly fewer larvae from colonies fed pollen-2 and reared in those colonies survived to the capped stage (*X*^2^ = 13.54, *p* < 0.0001) and emerged as queens compared with those reared in outside colonies (*X*^2^ = 4.21, *p* = 0.04). The percentage of larvae grafted from and reared in colonies fed pollen-1 that survived to the capped stage (*X*^2^ = 13.6, *p* < 0.0001) or emerged as queens (*X*^2^ = 10.0, *p* = 0.002) was significantly higher than when larvae were grafted from and reared in colonies fed pollen-2.

**Figure 4:**
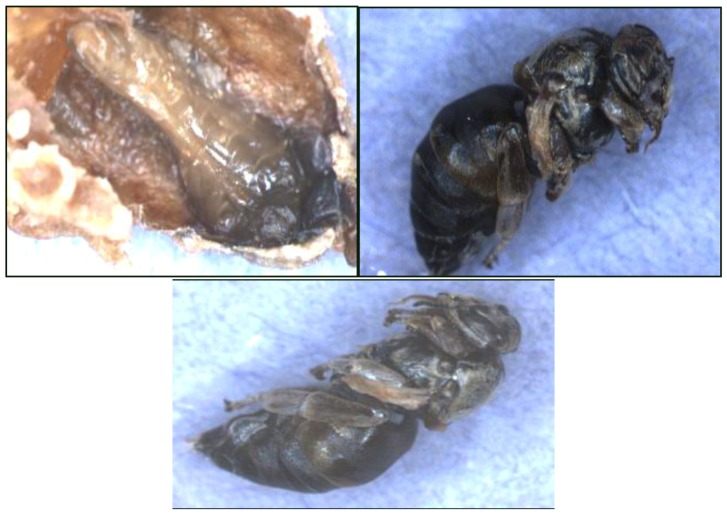
Examples of queen larvae from unemerged queen cells. The queens are representative of those that did not emerge when reared in colonies where pollen was contaminated with chlorpyrifos alone or with added fungicide (Pristine^®^).

When queen cells that did not emerge in both Experiments-1 and 2 were opened, we found that either the larvae did not successfully pupate and were a black viscous mass in the cell or were fully formed dark black pupa but were dead in the cells (Figure 4).

### 3.4. The Prevalence of Virus in Queens

All samples were tested for the presence and relative quantity of seven viruses, but only DWV and BQCV were frequently found. IAPV was detected only in colonies fed pollen-1 or 2.

**Figure 5:**
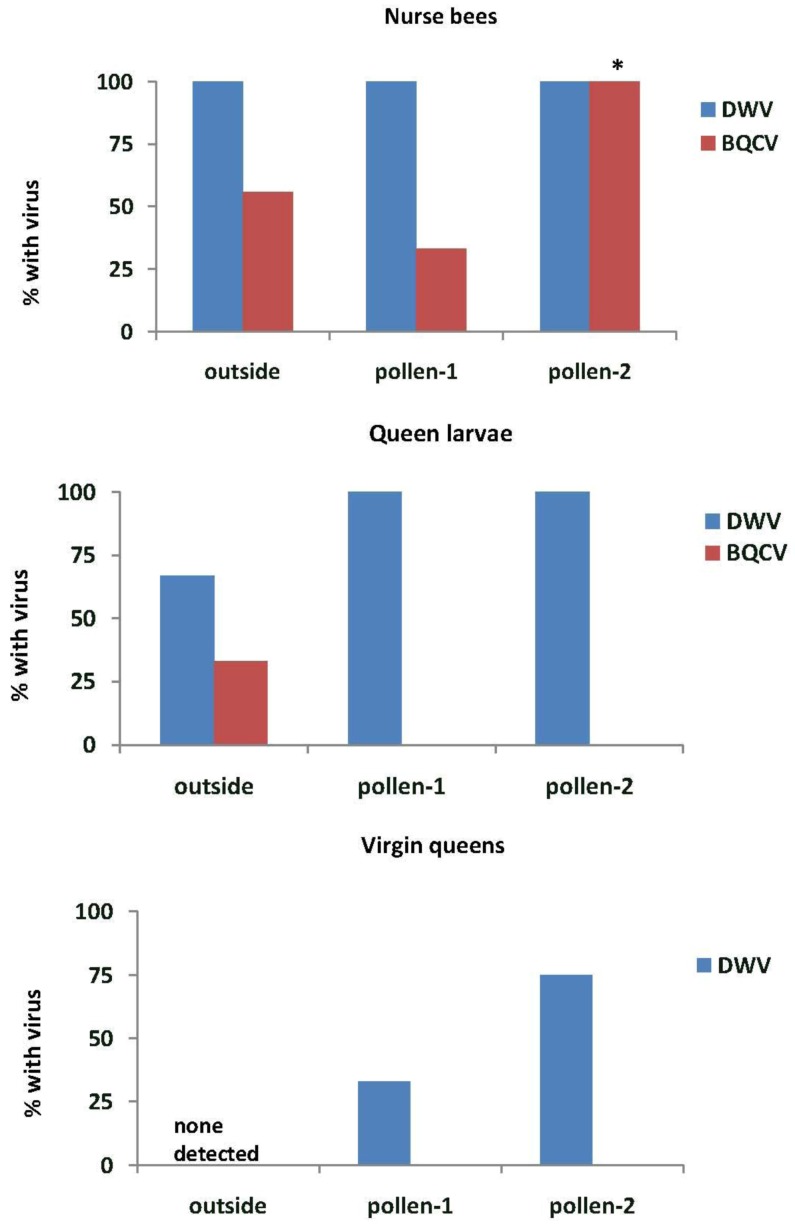
Nurse bees, queen larvae and virgin queens with deformed wing virus (DWV) and Black queen cell virus (BQCV). Samples were taken from open foraging colonies in an apiary (outside) or foraging in an enclosed flight are (EFA) and fed pollen with chlorpyrifos alone (pollen-1) or with added Pristine^®^ fungicide (pollen-2). Percentages of nurse bees with virus were based on samples of 9 nurse bees from outside colonies, 12 from colonies fed pollen-1 and 15 from those fed pollen-2. Nurse bees were sampled from those tending the queen cells. The percentages of queen larvae and virgin queens were analyzed based on three samples from colonies in each treatment group. Percentages with an asterisk are significantly different at *p* < 0.05 as determined by a Chi-square goodness of fit test.

In Experiment-1, DWV was found in all nurse bees tending the queen cells in outside colonies and in the EFA (pollen-1 and pollen-2) (Figure 5). All samples of royal jelly in the queen cells (n = 3 for each treatment group) also tested positive for DWV (data not shown). BQCV was detected more frequently in nurse bees from colonies fed pollen-2 than in outside colonies (*X*^2^ = 8.0, *p* = 0.018) or in those fed pollen-1 (*X*^2^ = 14.2, *p* = 0.001). All royal jelly samples from outside colonies had BQCV and 33% and 67% of those from colonies fed pollen-1 or 2 respectively. IAPV was detected only in nurse bees from colonies fed pollen-2 (13.3%), but it was present in 67% of the royal jelly samples from colonies fed pollen-1 and 33% of those fed pollen-2. DWV was found in most of the queen larvae reared in outside colonies and all of those reared in the EFA. BQCV was detected only in queen larvae reared in outside colonies. Some virgin queens reared in the EFA tested positive for DWV, but neither DWV nor BQCV were found in virgin queens reared in outside colonies.

**Figure 6:**
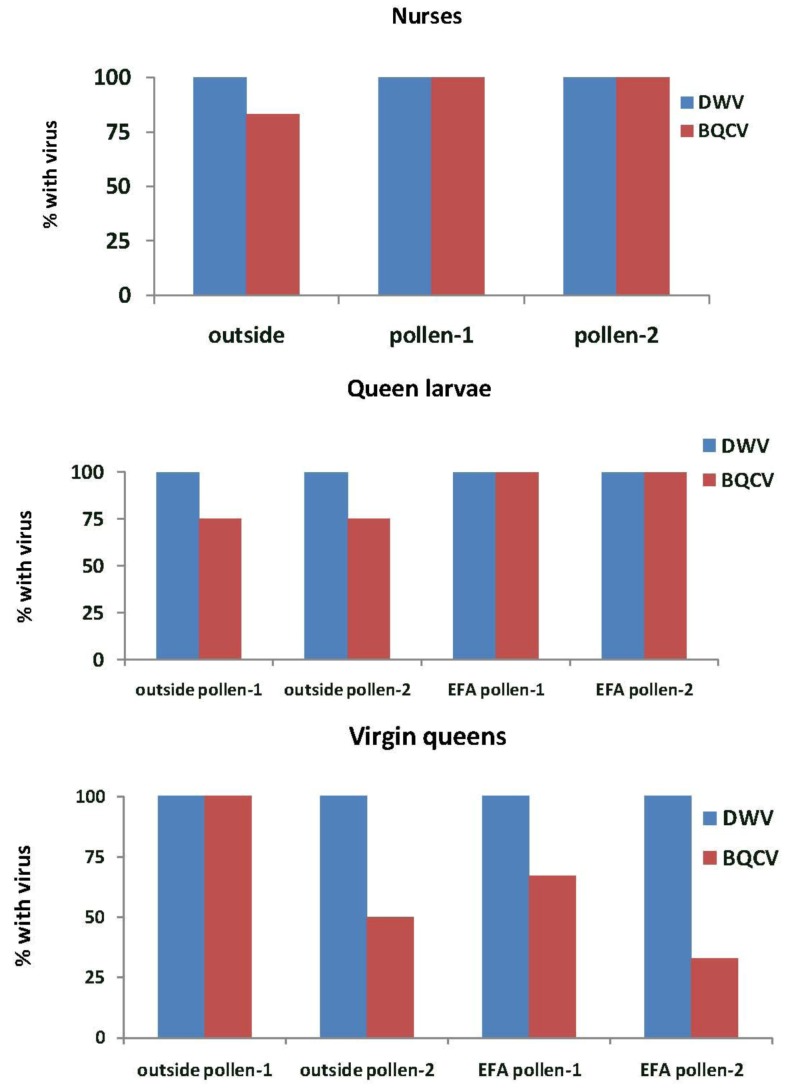
Nurse bees, queen larvae and virgin queens with deformed wing virus (DWV) and Black queen cell virus (BQCV). Samples were taken from open foraging colonies in an apiary (outside) or foraging in an enclosed flight are (EFA) and fed pollen with chlorpyrifos alone (pollen-1) or with added Pristine^®^ fungicide (pollen-2). The nurse bees were sampled while tending the queen cells containing the queen larvae that were tested. Percentages are based on samples of 12 nurse bees, three queen larvae, and three virgin from each treatment group. The exception was outside larvae reared in colonies fed pollen-2 where two virgin queens were analyzed.

In Experiment-2, DWV was detected in all nurse bees, queen larvae and virgin queens in the outside colonies and those in the EFA (Figure 6). BQCV was detected in 83% of the nurse bees in outside colonies and in all of those sampled in the EFA. These percentages were not significantly different (*X*^2^ = 2.18, *p* = 0.336). More than half of the queen larvae reared in outside colonies and all of those reared in the EFA had BQCV. All virgin queens grafted from colonies fed pollen-1 and half of those grafted from colonies fed pollen-2 that were reared in outside colonies had BQCV. The virus also was detected in 67% of the virgin queens grafted from and reared in colonies fed pollen-1 and 33% of those fed pollen-2.

**Figure 7:**
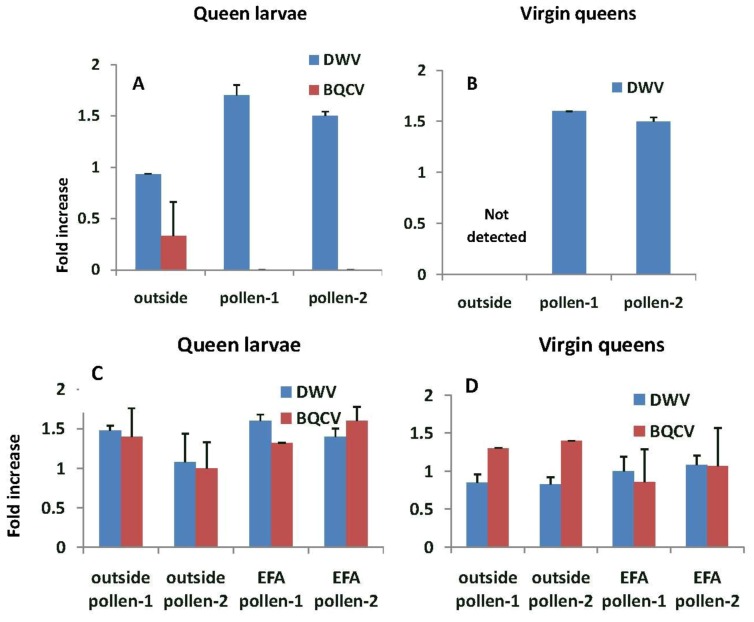
Fold increase in deformed wing virus (DWV) and Black queen cell virus (BQCV) titers relative to the nurse bees sampled while tending queen cells. (**A**) and (**B**) are from queens reared from grafts of larvae from outside colonies and reared in either outside colonies or those in an enclosed flight area fed pollen-1 (chlorpyrifos) or pollen-2 (chlorpyrifos with added Pristine^®^). (**C**) and (**D**) are from larvae grafted from and reared into queens in colonies fed pollen-1 or 2 or reared in outside colonies. Estimates of virus titers were made from 3 queen larvae and 3 virgin queens in each treatment group with the exception of outside larvae that were reared in colonies fed pollen-2 where two virgin queens were analyzed. Estimates of virus titers in nurse bees were based on samples of 9–15 bees per treatment group.

In general, fold increases of virus were higher when larvae were grafted from and reared into queens in EFA colonies compared with outside colonies (Figure 7). The prevalence of DWV in queen larvae grafted from and reared in outside colonies was similar to that of the nurse bees (*i.e.*, fold increase were close to 1.0) and significantly lower with respect to the fold increase, than in larvae being reared in pollen-1 or 2 colonies (F_2,5_ = 26.54, *p* = 0.002). We did not detect DWV or BQCV in virgin queens reared in outside colonies. In contrast, the prevalence of DWV and BQCV in queen larvae grafted from and reared in colonies fed pollen-1 or 2 was about 1.5 times higher than the nurse bees tending the queen cells. However, fold differences in DWV (F_3,10_ = 1.15, *p* = 0.377) and BQCV in queen larvae did not differ among the treatments (F_3,10_ = 0.88, *p* = 0.482) The prevalence of DWV and BQCV in the virgin queens were similar to or lower than the queen larvae. Fold differences in both DWV (F_3,7_ = 0.77, *p* = 0.548) and BQCV in emerged (virgin) queens did not differ among treatments (F_3,6_ = 0.39, *p* = 0.694; BQCV titers in outside larvae reared in pollen-2 colonies were not included in the analysis because only one queen tested positive for the virus).

## 4. Discussion and Conclusions

The emergence rates of queens and the virus titers they carried were affected by the presence of CPF alone and with added fungicide in pollen. Larvae either grafted from or reared as queens in colonies fed the contaminated pollen were less likely to emerge and more likely to test positive for both DWV and BQCV than those grafted from and reared in outside colonies. Protein concentrations in bee bread and the hemolymph of nurse bees were similar between colonies in the EFA and in outside colonies, therefore differences in queen emergence or virus titers could not be attributed to nutritional stress [25]. Though CPF alone appears to reduce queen rearing success, the reductions were greater when the fungicide also was present.

There was a progressive decline in the concentration of CPF and both components of Pristine^®^ relative to the pollen in the bee bread and nurse bees. The compounds were found in nurse bees probably because their guts contained the contaminated pollen and bee bread. Lower levels of pesticides in worker bees compared with the pollen in their colonies have been previously reported by Mullin *et al*. [10] and attributed to biotransformation to undetected or excreted metabolites. We did not detect the CPF or the components of Pristine^®^ in the royal jelly or queen larvae. Thus, the effects of the compounds on queen rearing and virus titers were not due to direct exposure of the queen larvae or their food. Rather, the reduction in queen emergence might have been due to transmission of virus through nurse bees and royal jelly into queen larvae. If immunity in the nurse bees was suppressed due to the contaminants, the virus titers being transferred might have been sufficient to cause death in the developing queens.

The differences in queen emergence rates between larvae reared in outside colonies and in the EFA indicate that both the larvae and the queen rearing environment were affected by the contaminants. The highest queen emergence occurred when larvae were grafted from and reared in the outside colonies. We did not detect virus in those emerged queens. However, if larvae from those same source colonies were reared in hives fed either pollen-1 or 2, only about 50% of them emerged and DWV was detected in some of them. The difference in queen emergence and virus titers between rearing environments might be partially attributed to greater stress in the EFA colonies compared with those outside where bees were open foraging. Though bees foraged in the EFA and collected both pollen and water, they were confined and could not fly as far from the hive as foragers from outside colonies. The bees in the EFA also could not collect resources directly from flowers as did the bees in open foraging colonies. Still. The contaminants in the pollen seemed to have an effect on queen rearing that was greater than differences in the location of the queen rearing colonies. For instance, when larvae were grafted from colonies in the EFA and reared in the outside colonies they had lower emergence rates and tested positive for virus with greater frequency than when larvae from outside colonies were reared in the same environment. Though the larvae selected for grafting were less than 36 h old, the effects of the contaminants were already present, and their chances of developing to the capped brood stage or emerging did not differ, in the case of larvae fed pollen-1, between those reared in pollen-1 or outside colonies. Pettis *et al*. [8] reported similar findings where pesticide exposure during the immature stages was associated with increased numbers of *Nosema* spores in pesticide-free adult bees.

The findings from this study suggest that queen producers should not use pollen in their queen rearing colonies that might be contaminated with pesticides and fungicides even if the worker bees seem to be unaffected. There could be severe reductions in queen cell capping and emergence. Furthermore, the queens that do emerge might have DWV and thus extend the impact of pollen contamination into the colonies headed by those queens. Though the effects of DWV on the queens themselves seem to be minimal, there is an association between DWV infection in ovaries and the degeneration of individual follicles [26]. More importantly though, DWV is transmitted vertically from queens to their offspring [27,28,29] resulting in a persistent latent infection circulating in the colony population. Under appropriate environmental or biological stress, the viruses become activated and cause various pathologies in the hosts. These include behavioral deficiencies [30], the typical deformed wing pathology, and significantly reduced life expectancy [31]. There also is a strong association between DWV in worker bees and colony mortality over winter [26,32,33,34,35]. Thus, the sublethal effects of pollen contamination might contribute to the pervasive presence of DWV in managed colonies and eventual colony loss when the viruses are activated by stress factors such as Varroa mites or the pesticide treatments used to control this parasite [36].

Though CPF alone affected queen emergence rates and virus titers, the impact was greater when combined with a Pristine^®^. The lowest rate of queen emergence occurred in pollen-2 colonies. The combination of a neurotoxicant (CPF) and an inhibitor of mitochondrial function (Pristine^®^) might have caused the care of the queen cells by nurse bees to be compromised so that the queen larvae were nutritionally stressed. This alone could have reduced the rate of cell capping and queen emergence. In addition, both CPF and the fungicide could have been affecting innate immunity in the nurse bees so that they were transmitting higher virus titers to the larvae. Indeed, virus titers in queen larvae reared in pollen-2 colonies were higher than in the nurse bees and there was higher mortality prior to the capped cell stage in this treatment. Thus, the effects of CPF that is a common contaminant of pollen might be amplified if it is present when Pristine^®^ is applied and the crop is in bloom.

Most investigations examining the effects of sublethal pesticide exposure on bees have focused on single compounds that bees are commonly exposed to in the field (e.g., see refs in [37]) or in the hive to control Varroa [36,38,39]. However, colonies are rarely exposed to single pesticides, and testing individual compounds might underestimate the impact on colony health and survival. The effects of fungicides on honey bee physiology also might be underestimated especially if these compounds affect the beneficial microbes bees use to preserve and digest their food especially pollen [40,41]. More accurate assessments of sublethal effects of pesticides might be made by examining them in combinations that colonies commonly encounter. It is the combinations that might ultimately affect the bees’ ability to metabolize food and survive in the presence of parasites and pathogens.
